# Uveitis outcome and complication

**DOI:** 10.1186/1546-0096-12-S1-I5

**Published:** 2014-09-17

**Authors:** Ivan Foeldvari

**Affiliations:** 1 Schön Klinik Hamburg Eilbek, Schön Klinik Hamburg Eilbek, Hamburg, Germany

## 

Anterior uveitis is a well-known threatening comorbid condition of JIA and affects around 10 to 20 % of the patients depending on JIA subtype. A large proportion of children with JIA develop uveitis in the first year of disease and 73 to 90% do so after four years of the arthritis onset. Uveitis can progress into the adulthood and usually occur as “white uveitis” which is not associated with symptoms like redness and pain as opposed to JIA related to the enthesitis subtype that is symptomatic. Factors associated to lower uveitis remission rate are: JIA diagnosis, findings of 1+ or more vitreous cells at presentation and initial visual acuity of 20/200 or worse. The Standardization of Uveitis Nomenclature (SUN) Group took the first step to define outcome measures for uveitis, but it was established just for adults. The Multinational Interdisciplinary Working Group for Uveitis in Childhood (MIWGUC) proposed outcome measures for JIA associated uveitis incorporating the Standardization of Uveitis Nomenclature (SUN) criteria in 2011.

## Disclosure of interest

None declared.

